# From an Incidental Finding to an Emergent Treatment: A Case Report of a Hepatic Adenomatosis and Large Ruptured Hepatic Adenoma

**DOI:** 10.1155/2021/9963440

**Published:** 2021-06-19

**Authors:** Maria Mironova, Mohammed K. Mahdi, Jyoti Bhatia, Rosemarie C. Nielson, Cataldo Doria

**Affiliations:** ^1^Department of Medicine, Capital Health Regional Medical Center, 750 Brunswick Ave, Trenton, NJ 08638, USA; ^2^Department of Gastroenterology, Capital Health Hopewell, One Capital Way, Pennington, NJ 08534, USA; ^3^Cancer Center, Capital Health Hopewell, Two Capital Way, Pennington, NJ 08534, USA

## Abstract

*Introduction*. Hepatic adenoma is an uncommon benign liver tumor presenting as solitary lesions or even rarely as hepatic adenomatosis. Large lesions carry a risk of rupture, hemorrhage, and malignant transformation. This case report aims to increase awareness about risk factors for hepatic adenomas, considering the increasing prevalence of obesity and the widespread use of oral contraceptive pills. *Case Presentation*. A 20-year-old obese female who was taking oral contraceptive pills for seven years presented to the emergency department with vomiting and abdominal pain caused by gastroenteritis. On imaging, multiple hepatic adenomas, including two lesions 6 and 9 cm in diameter, were incidentally found. During the hospitalization, the patient suddenly developed acute anemia and rupture of the largest lesion, which was promptly treated with arterial embolization. *Discussion*. Obesity and exposure to hormones are well-known risk factors for hepatic adenomas. The incidence of hepatic adenomas is steadily increasing because of the prevalence of obesity, especially among females. Lifestyle interventions for weight loss and discontinuation of oral contraceptive pills are considered a conservative treatment of hepatic adenomas. Large lesions possess the risk of malignant transformation and rupture and require surgical excision.

## 1. Introduction

Hepatocellular or hepatic adenomas (HAs) are benign tumors of presumable epithelial origin that occur in less than 0.004% of the population at risk [1, 2]. Their presentation varies from an asymptomatic solitary lesion, discovered incidentally, to hepatic adenomatosis, characterized by ten or more hepatic adenomas. Lesions bigger than 5 cm occur in less than 20% of cases and have a high potential for malignant transformation, spontaneous hemorrhage, and rupture. Exposure to hormones, including oral contraceptive pills (OCPs), and obesity have been strongly associated with HA occurrence. The risk of HA in women increases with the prolonged use of OCPs [3–5].

We present a case of an obese young female in whom hepatic adenomatosis was found incidentally. Three days after the discovery, the patient developed a rupture of the largest lesion and acute anemia, which led to an emergent, but prompt embolization and partial control of bleeding.

## 2. Case Presentation

A 20-year-old woman presented to the emergency department for the evaluation of the acute onset of epigastric and chest pain, shortness of breath, and vomiting that started the same day after eating a tuna sandwich. Her medical history was significant for hypothyroidism, depression treated with sertraline, and dysmenorrhea. She was taking OCPs for the past seven years. The body mass index was 34, vital signs were prominent for hypertension 164/81 mmHg, and tachycardia 99 bpm. A physical exam revealed palpable hepatomegaly. Laboratory data were remarkable for leukocytosis of 16,000/mm^3^ and transaminitis with aspartate transaminase of 267 and alanine transaminase of 333 U/l.

**Figure 1:**
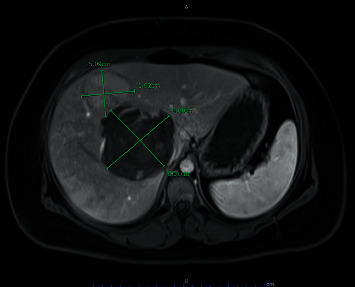
MRI of the liver with contrast showing a sizable hemorrhagic HA measuring 9.0 × 8.5 cm and adjacent nonhemorrhagic HA 5.9 × 5.1 cm (segment 4) and several HAs smaller than 5 cm in diameter.

The patient was tested for SARS-CoV-2 infection, considering the ongoing pandemic. The polymerase chain reaction test returned negative. The differential diagnosis also included viral hepatitis, viral or food-borne gastroenteritis, and pulmonary embolism, considering the history of prolonged hormonal treatment. Computerized tomographic (CT) angiography of the chest was negative for pulmonary embolism but noted multiple large hyperenhancing liver masses. The patient was diagnosed with gastroenteritis, and symptomatic treatment was continued. However, the incidental finding of liver masses prompted further investigation. Blood work, including viral hepatitis panel, carcinoembryonic antigen, and cancer antigens 19–9 and 125, were within normal limits. The magnetic resonance imaging (MRI) of the liver was performed to differentiate between possible liver neoplasms, abscesses, hemangiomas, and adenomas. It showed hepatic steatosis and multiple masses involving both lobes. The MRI findings indicated that one of those lesions was an inflammatory hemorrhagic lesion of the hepatic dome, measuring 9.0 × 8.5 cm with severe mass effect and flattening of the intrahepatic inferior vena cava. Another one was a nonhemorrhagic lesion 5.9 × 5.1 cm, inseparable from the dominant mass (Figure 1). There were multiple other nonhemorrhagic HAs smaller than 4.5 cm. The diagnosis of hepatic adenomatosis was postulated as the patient was taking OCPs over a prolonged period. Contraceptives were discontinued, and weight loss was recommended.

**Figure 2:**
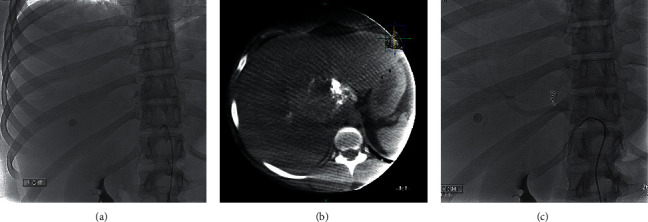
(a) Subselective catheterization of the hepatic artery, segment 8 branch with arteriography. (b) Cone beam CT demonstrating patency and perfusion of the hepatic adenoma. (c) Coil embolization of segment 8 branch of the hepatic artery.

**Figure 3:**
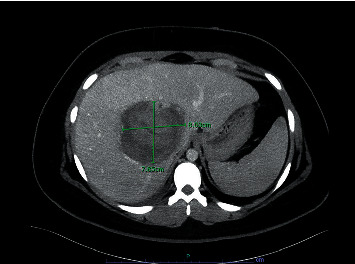
CT scan of the abdomen performed the next day after embolization showing the decrease in the dominant lesion size to 8.0 × 7.9 cm.

After three days at the hospital, the patient developed acute anemia with a drop in the hemoglobin level from 11.5 to 8.7 g/dL. The CT scan of the abdomen showed an increase of the largest adenoma to 9.5 cm in diameter, thus confirming the rupture. The emergent transarterial embolization of the lesion was performed, considering rapid expansion and life-threatening hemorrhage risk (Figure 2). The CT scan completed the next day showed a decrease of adenoma to 8.0 cm × 7.9 cm (Figure 3) along with the increase in hemoglobin levels, confirming the successful embolization.

**Figure 4:**
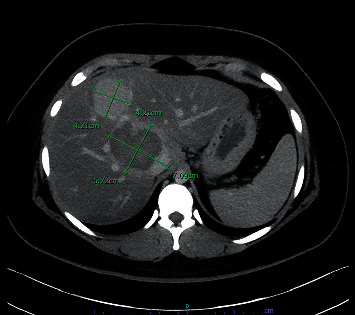
CT scan of the abdomen repeated in two months showing that the dominant HA decreased in size, measuring 7.1 × 5.7 cm, with coils and a resolved hemorrhagic component. The nonhemorrhagic mass in segment four decreased to 4.2 cm in diameter.

The patient had a follow-up CT scan of the abdomen two months after embolization. It showed a decrease in the size of the dominant lesion with a complete resolution of the hemorrhagic component (Figure 4). The option of resection of this lesion was discussed with the patient.

## 3. Discussion

Exposure to hormones, including OCPs and anabolic steroids, is a known risk factor for HA. It was proven that the use of OCPs for five to seven years carries a 5-fold risk of HA, and after nine years, the risk becomes 25-fold [6]. The injury mechanism is associated with inhibition of bilirubin and bile secretion by estrogens and induction of cholestasis. Discontinuation of hormonal treatment and close monitoring is considered conservative management of HA. Usually, it leads to a regression in tumor size, at least by one-third in 40% of the patients [7, 8].

The modern-generation OCPs are safer and have less impact on the risk of HA. However, the incidence of HA has been steadily growing, driven by the increasing number of overweight and obese female patients [9]. Also, patients with a higher BMI usually develop multiple and inflammatory adenomas, as was seen in this case. Weight loss is strongly encouraged for individuals with HA. Recent studies show that bariatric surgery is associated with tumor regression or even complete resolution [10].

The definite diagnosis of the nature of adenomas is usually made by performing a biopsy of the lesions. In our case, a biopsy was not performed; however, the MRI identified early arterial enhancement without portal washout and a hyperintense signal band on the periphery of the lesion. That finding was strongly suggestive of the inflammatory nature of the adenomas in the patient [11].

HA can result in spontaneous hemorrhage in 20% of the cases and malignant transformation to hepatocellular carcinoma in 5% [1]. The risk of these complications increases with the size of the lesion. Most of the hemorrhages are intratumoral, but in case of massive bleeding, the lesion can rupture, leading to a subcapsular hematoma [12]. Symptomatic lesions or size larger than 5 cm are indications for surgical resection or selective arterial embolization in case of rupture or hemodynamic instability [13]. Radiofrequency ablation can be used in the case of multiple HA, usually requiring several sessions [14, 15].

In conclusion, the combination of hemodynamic compromise and obesity or the history of exposure to hormones in a female should always raise suspicion for the possibility of a ruptured HA and prompt the necessary workup and management.
